# Persistent Emergence of Dengue

**DOI:** 10.3201/eid1105.050195

**Published:** 2005-05

**Authors:** Charles H. Calisher

**Affiliations:** * Colorado State University, Fort Collins, Colorado, USA

**Keywords:** dengue, emergence, epidemic, DHF, vaccines, prevention

The disease dengue fever (DF; also known as breakbone fever, dandy fever, and by other names) can be caused by any of 4 viruses within the virus family *Flaviviridae*, genus *Flavivirus*, i.e., dengue virus types 1–4 (DENV-1–4). Dengue fever is a short-duration, nonfatal illness characterized by sudden onset of headache, retroorbital pain, high fever, joint pain, and rash. Whereas uncomplicated DF usually is the case, the picture can be much darker than that. Through a mechanism known as immune enhancement, sequential infections with certain dengue viruses set the stage for a far more serious complication, dengue hemorrhagic fever (DHF) and dengue shock syndrome, so that having uncomplicated DF can presage having DHF (1).

DHF is characterized by high fever, vascular permeability, bleeding, enlargement of the liver, and circulatory failure (dengue shock syndrome). In mild or moderate cases, signs and symptoms subside after the fever subsides, but in severe cases the patient's condition suddenly deteriorates, body temperature decreases, and the circulatory system begins to fail. The patient then may quickly go into shock and die within a day, or quickly recover, if volume therapy is instituted (*2*).

Dengue viruses are transmitted from person to person or from monkey to monkey through infected female mosquitoes of the genus *Aedes*. The mosquito acquires the virus by taking a blood meal from an infected human, the principal amplifying host for these viruses, or from an infected monkey. Humans circulate these viruses in their blood (viremia) for 7 to 10 days after infection, allowing ample time for mosquitoes, often many mosquitoes, to feed and become infected. After an intrinsic incubation period of 1 week to 10 days, the mosquito is capable of transmitting the virus to a new host while blood feeding.

During epidemics of dengue, attack rates may be 80%–90% in susceptible persons. Although, it is not usually recognized, more than half the people who are infected with a dengue virus may be asymptomatic, which would indicate a substantial underreporting of infections. These comprise a substantial number of people who may have been primed for more serious illness at a later date and are unaware of their situation.

The global prevalence of dengue has increased substantially recently. Dengue is endemic in ≈100 countries in Southeast Asia, Africa, the Western Pacific, the Americas, Africa, and the eastern Mediterranean area (available from http://www.who.int/mediacentre/factsheets/fs117/en/), with imported cases essentially everywhere tourists, business people, and military personnel travel, whether dengue is recognized there or not. More than 2 billion of the approximately 6.5 billion inhabitants of this planet are at risk of acquiring dengue, and the World Health Organization has estimated that "there may be 50 million cases of dengue infection worldwide every year" (available from http://www.who.int/mediacentre/factsheets/fs117/en/). However, this is a misstatement. Either there are 50 million dengue infections (some with illness, some not) or there are 50 million people sick with dengue each year. Infections are not the same as illnesses.

The mild form of dengue is a serious annoyance and often is painful for those with it. However, DHF is the major international public health concern. Before 1970, a total of 9 countries had reported DHF epidemics; by 1995, >4 times that number reported such outbreaks. Most of these countries are in Southeast Asia and the Western Pacific, but with the worldwide spread of all dengue types, this disease threatens residents in tropical and subtropical regions, predominantly in urban and semiurban areas. In 2001, ≈600,000 cases of dengue were reported in the Americas, of which 15,000 were cases of DHF, more than twice the number of DHF cases in the Americas in 1995 (available from http://www.who.int/mediacentre/factsheets/fs117/en/). In 2001 alone, Brazil reported nearly 400,000 cases, including 670 cases of DHF. Not only is the geographic distribution of dengue spreading, but the seriousness of its complications is being recognized (available from http://www.cdc.gov/search.do?action=search&queryText=dengue). An estimated 500,000 persons with DHF require hospitalization each year, a substantial proportion of whom are children. Tragically, DHF is a leading cause of hospitalization and death of children in several Asian countries. Case-fatality rates can exceed 20%, are usually 2.5%, but can be reduced to <1% with rapid recognition and proper treatment.

In countries that are prepared for dengue and its complications, diagnostic services are available. We are able to sequence these viruses and determine their origins and evolutionary determinants. We can, to some degree, control the vector mosquitoes (*Aedes aegypti* and *Ae. albopictus*). Our knowledge of the pathophysiology of DHF is quite sophisticated (*2**,**3*). A vast literature is available about these viruses and the diseases they cause. Why, then, does dengue continue to spread? If we cannot eradicate dengue (and its vector *Ae. aegypti*) from populations on islands, from where can we eradicate it? Politics or misdirected funding, as always, has something to do with this, but the situation is much more complicated than that. Unless transovarial transmission (passage of virus from female to offspring through the egg) is much more important than it appears to be, other mechanisms are at play. Univalent vaccines for these viruses have been prepared but, for the most part, health authorities are (justifiably) unwilling to use such vaccines because they have the potential to stimulate the production of antibodies, which would prime vaccinees for DHF by immune enhancement. Fortunately, novel approaches (development of incompetent mosquitoes), development of modern tetravalent vaccines, and development of chimeric vaccine viruses (*4*), using classic as well as molecular approaches will soon be available and hold out promise of tools we need to eliminate or eradicate this scourge.

This issue of Emerging Infectious Diseases includes some very interesting reports on dengue and its clinical complications, dengue diagnosis, and dengue epidemiology. These add considerably to the scientific record.

**Figure:**
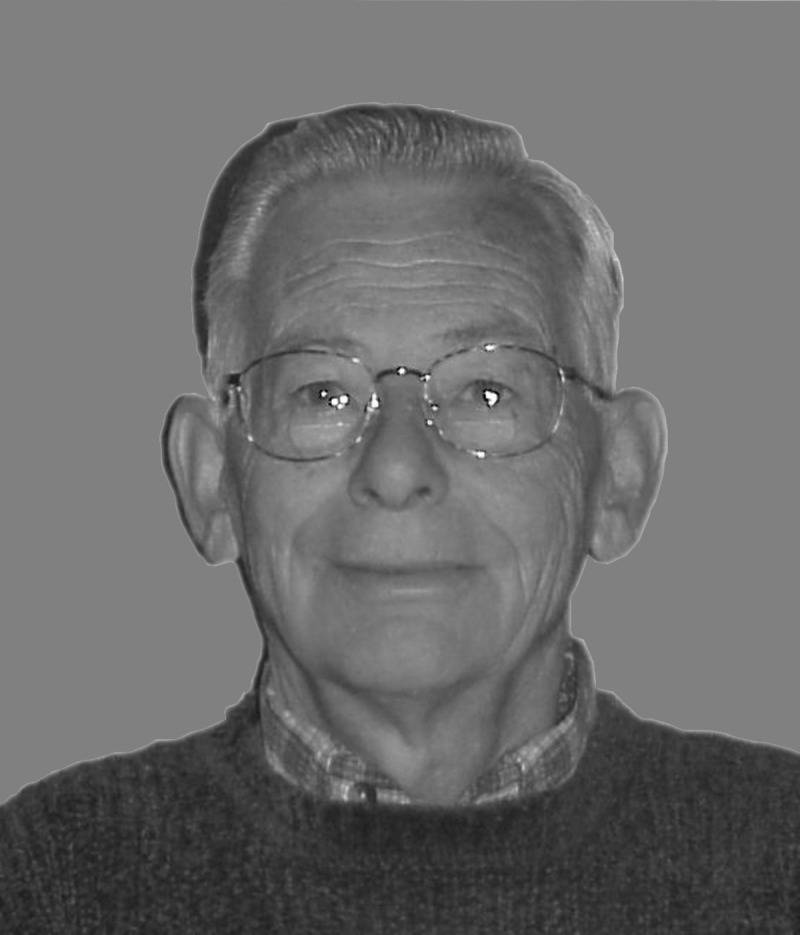
Dr. Calisher is professor of microbiology in the Arthropod-borne and Infectious Diseases Laboratory, Department of Microbiology, Immunology, and Pathology, Colorado State University. His interests include arboviruses and the diseases they cause, the biology of arthropod vectors of viruses, rodent-borne viruses and the diseases they cause, and the epidemiology of viruses.
